# Fear of disease progression among breast cancer patients in China: a meta-analysis of studies using the fear of progression questionnaire short form

**DOI:** 10.3389/fpsyg.2023.1222798

**Published:** 2023-08-23

**Authors:** Jia-Li He, Hui-Qiong Xu, Jing Yang, Dong-Jiang Hou, Xiao-Yan Gong, Xian-Ying Lu, Wei Wang, Ming-Jin Cai, Yu-Feng Yu, Jing Gao

**Affiliations:** ^1^School of Nursing, Chengdu University of Traditional Chinese Medicine, Chengdu, Sichuan, China; ^2^Division of Abdominal Tumor Multimodality Treatment, Cancer Center, West China Hospital, Sichuan University, Chengdu, Sichuan, China; ^3^West China School of Nursing, Sichuan University. Chengdu, Sichuan, China; ^4^School of Medicine and Life Science, Chengdu University of Traditional Chinese Medicine, Chengdu, Sichuan, China

**Keywords:** breast cancer, fear of disease progression, prevalence, meta-analysis, systematic review

## Abstract

**Background:**

Fear of disease progression (FoP) is among the most prevalent and major psychological burdens breast cancer patients encounter. Excessive FoP may result in serious adverse effects for patients. FoP in breast cancer patients has gained attention recently; however, its prevalence in China is unknown.

**Objectives:**

This meta-analysis and systematic review aimed to assess the overall FoP among Chinese breast cancer patients to make recommendations for treatment and care.

**Methods:**

Systematic search databases included PubMed, EMbase, The Cohrane Library, Web of Science, CINAHL, PsycINFO and 4 Chinese databases (Wan Fang Data, CBM, VIP and CNKI). The retrieval time ranged from the database’s establishment to March 20, 2023. After two researchers independently evaluated the literature, retrieved information, and assessed the risk of bias for the included literature, Stata 15.1 software was used to conduct a meta-analysis.

**Results:**

A total of 37 moderate or high-quality studies involving 9,689 breast cancer patients were included. Meta-analysis showed that the pooled mean score of FoP for Chinese breast cancer patients was 33.84 [95% *CI* (31.91, 35.77)], prediction interval (21.57 ~ 46.11). The subgroup study found that FoP levels varied among breast cancer patients of different regions, ages, educational levels, marital statuses, residences, illness stages, and disease statuses.

**Conclusion:**

Breast cancer patients have higher FoP scores. Healthcare workers should be concerned. We expect that more relevant research will be undertaken and more effective interventions will be developed. Patients can manage their illness and improve their quality of life by reducing their fears.

**Systematic review registration:**

https://www.crd.york.ac.uk/prospero/, identifier: PROSPERO CRD42023408914.

## 1. Introduction

Breast cancer is the most common malignant tumor in women, and its prevalence is rising yearly. According to the most recent data, breast cancer has surpassed lung cancer as the most prevalent malignant tumor among women. Globally, there are 2.3 million new cases of breast cancer and 700,000 deaths each year (Sung et al., 2021). China has doubled the global rate of breast cancer growth (Du, 2019). In 2020, 18.4% of new breast cancer cases and 17.1% of fatalities were Chinese (Cao et al., 2021). Given the severity and persistence of the disease, the question of survival care for Chinese breast cancer patients is critical. Due to greater public health awareness, early detection, and medical technology advancement, breast cancer survival rates and times have improved (Hummel et al., 2015; He et al., 2021). However, disease recurrence and metastasis have remained a challenge for modern medical science and a source of worry and threat for cancer patients.

Fear of disease progression (FoP) was illustrated by German scholar Dankert (Dankert et al., 2003) in 2003 as fear of various bio-social-psychological repercussions of disease advancement or fear of disease recurrence. A systematic review indicated that cancer patients’ greatest unmet demand was fear about recurrence and progression (Lisy et al., 2019). Fear of disease recurrence and progression is one of the most common psychological responses to breast cancer and one of the most unmet psychosocial needs of oncology patients (Peng et al., 2019; Chen et al., 2020). However, research has revealed that, of all cancer types, FoP is the most common and persistent in breast cancer patients (Götze et al., 2019). A study by Reed (Reed et al., 2020) on 1,032 patients with various oncological cancers in the United States revealed that 73% of female breast cancer patients reported varying degrees of fear of disease progression. This percentage was significantly higher than that of patients with prostate cancer and gynecological tumors. Moreover, in breast cancer patients, FoP can last up to 16 years after diagnosis (Koch-Gallenkamp et al., 2016).

Unlike anxiety and depression, FoP is a definite, distinct, and actual fear that the patient experiences. It is usually somewhere between “functional” and “dysfunctional.” Low levels of FoP are a normal, transient emotional response to cancer that can help alert patients to the disease’s recurrence and motivate them to adopt better lifestyles (Simard and Savard, 2015). When FoP becomes a clinical issue, the patient’s physical, mental, and spiritual health may suffer. Patients often obsess over bodily symptoms, including hypervigilance, examinations, and comfort-seeking, which lowers their quality of life (Cho and Park, 2017). Furthermore, excessive FoP frequently leads patients to take on negative ways of coping with the disease, reducing treatment compliance. This seriously impacts patients’ ability to cope with illness, actively cooperate with cancer-fighting treatment effectively, and is not conducive to disease recovery (Yang et al., 2018). High FoP breast cancer survivors, may use medical services excessively due to stress and worry, increasing healthcare costs (Thewes et al., 2012; Dinkel and Herschbach, 2018). According to a review, when excessive medical practices connected to FoP are sensibly handled, they may have large economic advantages (Williams et al., 2021).

Based on the findings above, rapid assessment of FoP levels in breast cancer patients and appropriate therapies are needed to reduce physical and psychological stresses and promote efficient healthcare resource usage. Previous research has revealed that factors like cultural origins and disparities in national healthcare structures may influence the FoP of breast cancer patients (Ashing et al., 2017). Findings from one country cannot be extrapolated to other countries or areas, and it is also uncertain whether existing programs and interventions aimed at reducing FoP are appropriate or helpful for Chinese breast cancer survivors. As more foreign studies have been conducted, China’s academic and medical communities have better grasped FoP in breast cancer patients. However, research on FoP in cancer survivors began later in China than in Western countries like the Netherlands and the United States (Hui, 2021). The fear of progression questionnaire short form (FoP-Q-SF) (Mehnert et al., 2006) is the most commonly utilized in research examining FoP in Chinese cancer patients. Since Cai (Cai and Jiang, 2018) applied it to Chinese breast cancer patients in 2018, the number of therapeutically relevant research employing FoP-Q-SF to estimate the prevalence of FoP has gradually increased. Unfortunately, study results vary widely. For instance, Maio et al. (2020) study found reduced FoP levels in breast cancer survivors. However, other research has achieved different results, with Li et al. (2019) and Song (2018) study indicating that breast cancer patients had high levels of FoP and should be intervened as soon as feasible. Studies have not yet been able to systematically identify the characteristics of FoP prevalence in Chinese breast cancer patients due to budget and labor limitations. They cannot thoroughly analyze the issue for the entire nation.

Understanding FoP prevalence in Chinese breast cancer patients may help national policymakers and healthcare professionals make better recommendations and give crucial background information for improved mental health assessment and services. Thus, this research summarizes FoP levels in Chinese breast cancer patients and provides theoretical support for regulating FoP levels to improve patients’ quality of life and mental health. Meanwhile, it is an example for other nations.

## 2. Methods

### 2.1. Design

This systematic review was conducted under the guidelines of the Preferred Reporting Items for Systematic Reviews and Meta-Analyses (PRISMA) (Page et al., 2021) and was registered in PROSPERO website [CRD42023408914].

### 2.2. Literature inclusion and exclusion criteria

Literature inclusion criteria: (1) Populations: Patients with Chinese breast cancer diagnosed at any pathological stage, (2) Study type: Used observational studies (cohort, case–control and cross-sectional study), and (3) Outcomes: FoP-Q-SF was used to assess the FoP score in breast cancer patients.

Exclusion criteria: (1) adjustments were made to the FoP-Q-SF, and its total score was not consistent with the original scale, (2) mixed population of cancer patients, and information on breast cancer patients could not be clearly extracted, (3) non-English and Chinese literature, (4) if the literature was repeatedly published, the one with the most complete information was included, and (5) full text was not available or low-quality studies.

### 2.3. Search Strategy

A systematic search was conducted for studies published in PubMed, EMbase, The Cohrane Library, Web of Science, CINAHL, PsycINFO, Wiley, WanFang Database, Chinese Biomedical Database (CBM), Chinese Science and Technology Resource Integrated Database (CNKI), and Chinese Science and Technology Periodicals (VIP) database, with dates ranging from inception until 20 March 2023. The final retrieval strategy was formulated by combining MeSH terms and related keys (see Appendix A). In addition, the references of relevant studies were manually screened to identify other studies.

### 2.4. Study screening and data extraction

The literature was managed with EndnoteX9 software. After duplicates had been removed, two researchers (HJL and HDJ) independently examined the title and abstract to screen the literature before thoroughly reviewing the studies to decide which ones should be included. Using Microsoft Excel, two reviewers (HJL and YJ) independently extracted information. They then double-checked their results and, in cases of disagreement, consulted a third researcher (YYF or GJ). After reading the full text, the step was marked as completed. The data extraction content included the first author, year of publication, region, sample size, sample source, age, and the score of Fop (e.g., mean score, standard deviation). If the data are incomplete or missing, the author will be contacted by e-mail for information. Finally these information would be integrated and verified by the two researchers (XHQ and GXY).

### 2.5. Quality evaluation of the included studies

All included articles were cross-sectional studies, therefore, we used the Agency for Healthcare Research and Quality (AHRQ) to assess methodological quality (Zeng et al., 2015). The rating scale consists of 11 items, with a 1 for “yes” and a 0 for “no” or “unclear.” The assessments were classified as low, middle and high if the total scores were0 ~ 3, 4 ~ 7, 8 ~ 11, respectively. The assessment of quality was conducted by the reviewer (LXY) and double-checked by another reviewer (WW), discrepancies were resolved by discussion.

### 2.6. Meta-analysis

Quantitative data were integrated using meta-analysis because all trials used uniform measuring tools. By Stata 15.1, average Fop scale scores and standard deviations from several research were presented as weighted effect sizes and 95% confidence intervals (*CI*). The I^2^ test and Q test were used to analyze heterogeneity. When *I*^2^ ≤ 50% and *p* ≥ 0.1, a fixed-effects model was selected to combine effect sizes; in the opposite case, a random-effects model was selected. We also estimated the 95% prediction interval (PI), a PI was calculated based on the methods provided by Borenstein et al. (2017), showing the range of a true score of FoP of a future study in 95% of all patients. We also used meta-regression and subgroup analysis to investigate the causes of high heterogeneity. Moreover, sensitivity analysis was conducted by omitting an individual study each time and repeating the analysis to assess each study’s influence on the pooled effect size. Publication bias was evaluated by Egger’s test, and a value of p greater than 0.05 implied no publication bias. If publication bias existed, the trim-and-fill method was employed to detect the effects of publication bias on the results.

## 3. Results

### 3.1. Study selection process and results

**Figure 1:**
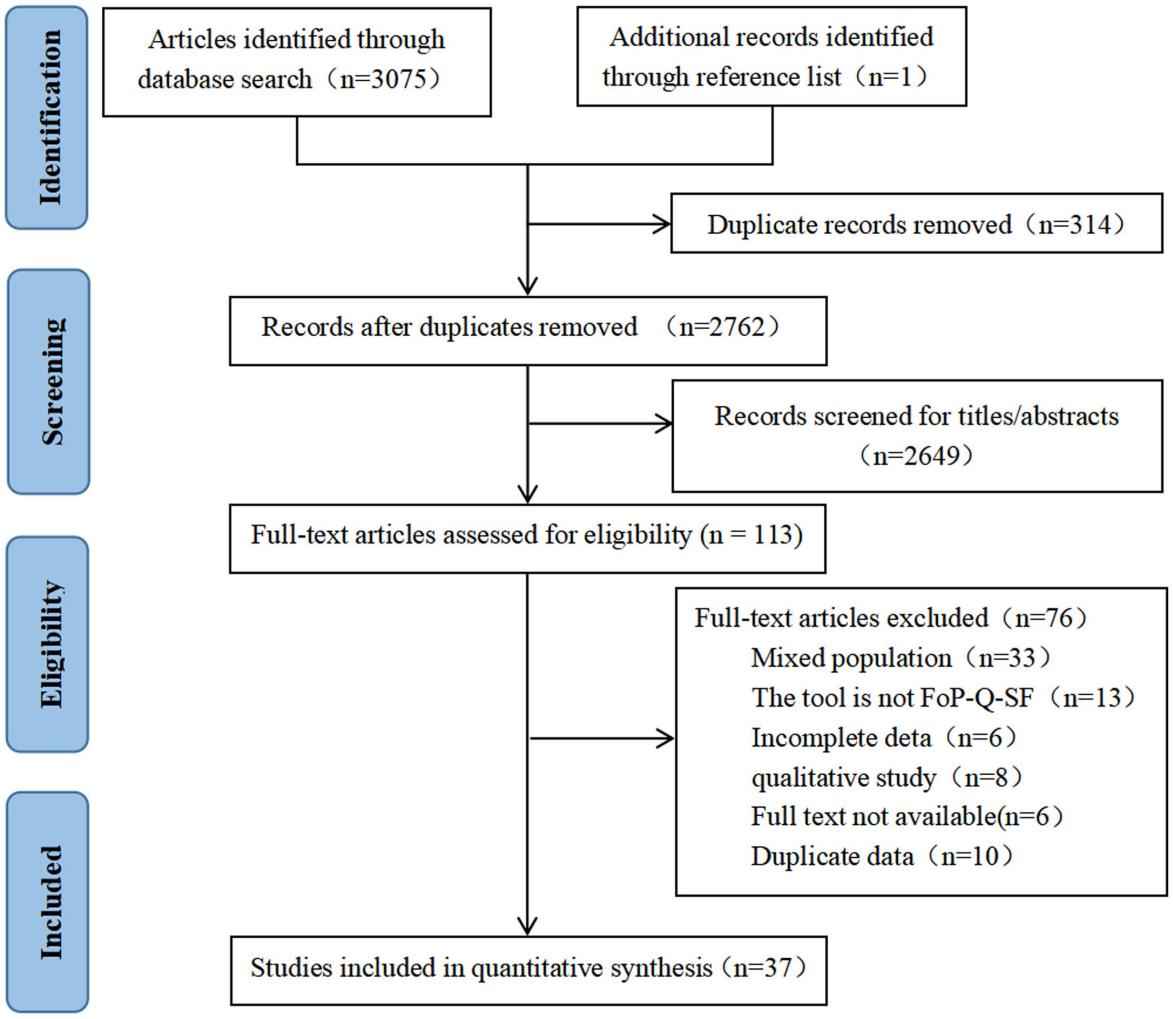
PRISMA flowchart of literature retrieval and selection.

3,075 records were retrieved from 10 databases, and and 1 were obtained by scanning relevant references, for a total of 3,076 records. After eliminating duplicate literature, the remaining studies were screened according to the title and abstract, and finally, 113 studies were included for full-text evaluation. Finally, a total of 37 articles were included in the systematic review and meta-analysis. The PRISMA flow diagram is shown in Figure 1.

### 3.2. Study characteristics

The review included 37 articles, all of which were cross-sectional studies, and 7 contained breast cancer patients who were the results of multicenter studies. All the articles were published in 2018 ~ 2022.

This study included 9,689 breast cancer patients aged 36.71 to 59.12 years. The Fop scores ranged from 23.57 to 45.79. Table 1 shows the fundamental traits of the included studies and the mean and total scores for each FoP-Q-SF scale dimension.

**Table 1:** Characteristics of included studies.

Author	Year	Region	Sample	Study design	Source of sample	Age Mean(SD)	Mean			Quality of study
							FoP	PH	SF	
Bao, and MI, Y. (2021)	2021	Shan Xi	274	cross-sectional	monocentric	58.47 (13.22)	45.79	NR	NR	6
Chen and Jiang (2020)	2020	Zhe Jiang	192	cross-sectional	monocentric	NR	27.09	16.89	14.18	7
Du et al. (2022)	2022	Shan Xi	203	cross-sectional	multicentric	50.08 (9.96)	36.80	NR	NR	8
Guo et al. (2022)	2022	He Bei	325	cross-sectional	monocentric	NR	38.79	18.22	16.84	8
He (2022)	2022	Tian Jin	246	cross-sectional	monocentric	50.19 (12.70)	36.53	16.6	14.5	6
Hou (2020)	2020	He Nan	510	cross-sectional	monocentric	NR	41.50	NR	NR	5
Hu et al. (2019)	2019	Zhe Jiang	124	cross-sectional	monocentric	51.10 (9.76)	24.92	18.05	6.87	7
Jia (2022)	2022	He Nan	346	cross-sectional	multicentric	NR	37.14	18.89	17.01	7
Jiang et al. (2020)	2020	Jiang Xi	110	cross-sectional	monocentric	57.23 (6.71)	38.54	16.01	12.68	6
Li et al. (2019)	2019	Shan Dong	364	cross-sectional	monocentric	NR	31.07	NR	NR	7
Li and Cheng (2020)	2020	Hong Kong	311	cross-sectional	multicentric	51.5 (12.3)	33.68	NR	NR	6
Li et al. (2022)	2022	He Nan	282	cross-sectional	monocentric	44.68 (8.38)	28.98	18.78	19.21	8
Lu et al. (2021)	2021	Shan Dong	160	cross-sectional	monocentric	NR	23.57	15.64	13.16	8
Lyu (2021)	2021	He Nan	230	cross-sectional	monocentric	NR	30.50	19.99	16.75	6
Lyu et al. (2020)	2020	Zhe Jiang	237	cross-sectional	monocentric	47.57 (9.83)	24.83	18.66	15.02	8
Maio et al. (2020)	2020	Bei Jing	103	cross-sectional	monocentric	NR	34.61	11.93	11.64	6
Niu et al. (2019)	2019	Jiang Su	342	cross-sectional	monocentric	51.46 (10.50)	30.72	21.89	16.9	8
Niu (2022)	2022	Ji Lin	257	cross-sectional	monocentric	52.38 (9.16)	28.79	17.49	12.77	8
Pan et al. (2022))	2022	Shan Dong	769	cross-sectional	monocentric	NR	36.73	NR	NR	7
Shan et al. (2022)	2022	Jiang Su	96	cross-sectional	monocentric	NR	33.96	NR	NR	7
Song (2018)	2018	Zhe Jiang	420	cross-sectional	multicentric	NR	29.24	23.52	22.26	7
Wang et al. (2021)	2021	Hu Nan	271	cross-sectional	monocentric	NR	37.24	15.27	15.45	6
Wang (2021)	2021	He Nan	64	cross-sectional	monocentric	NR	35.06	18.25	15.22	6
Wang (2021)	2021	Zhe Jiang	150	cross-sectional	monocentric	49.15 (8.93)	37.99	15.75	13.49	8
Wang et al. (2022)	2022	An Hui	293	cross-sectional	monocentric	52.1 (11.67)	26.82	18.07	17.82	6
Wang and Zhu (2022)	2022	He Nan	334	cross-sectional	monocentric	36.71 (6.72)	37.17	18.92	17.35	7
Wu et al. (2021)	2021	Shanxi	154	cross-sectional	monocentric	NR	28.62	18.45	18.75	7
Xie et al. (2021)	2021	The Yangtze River Delta/Shanxi/Guang Dong	488	cross-sectional	multicentric	NR	31.11	NR	NR	7
Xin (2022)	2022	Shan Dong	88	cross-sectional	monocentric	NR	35.91	NR	NR	7
Xing et al. (2018)	2018	Bei Jing	300	cross-sectional	monocentric	NR	35.89	NR	NR	6
Ye et al. (2019)	2019	Guang Dong	180	cross-sectional	multicentric	NR	30.26	NR	NR	7
Zhang et al. (2018)	2018	He Nan	270	cross-sectional	monocentric	NR	36.27	19.3	17.5	7
Zhang et al. (2019)	2019	He Nan	200	cross-sectional	multicentric	37.51 (4.50)	36.92	18.78	17.75	7
Zhang and Zhang (2019)	2019	Hu Nan	312	cross-sectional	monocentric	NR	37.54	NR	NR	7
Zhang and Duan (2022)	2022	He Nan	338	cross-sectional	monocentric	59.12 (11.23)	40.89	19.77	18.46	8
Zhang (2022)	2022	Shan Dong	227	cross-sectional	monocentric	47.7 (9.10)	32.83	17.63	15.21	8
Zhu et al. (2022)	2022	He Nan	119	cross-sectional	monocentric	NR	37.60	NR	NR	7

NR: Not reported; SD: standard deviation; FoP: Fear of disease progression; PH: physiological health; SF: Social/family.

### 3.3. Assessment of quality

The 37 included articles’ bias risk was evaluated, and the results revealed that the quality of the included studies was medium to high. The majority of the studies (*n* = 27, %) were classified as medium quality, whereas the remaining 10 studies (45.8%) were classified as high quality. Ten articles received an AHRQ score of 8 (high quality), while the remaining 27 received an AHRQ score of 5 to 7 (medium quality). The findings of the quality evaluation of the included studies are presented in Table 1, and the details of the evaluation process are presented in Appendix B.

### 3.4. Meta-analysis of the results

#### 3.4.1. Fear of disease progression of breast cancer patients

**Figure 2:**
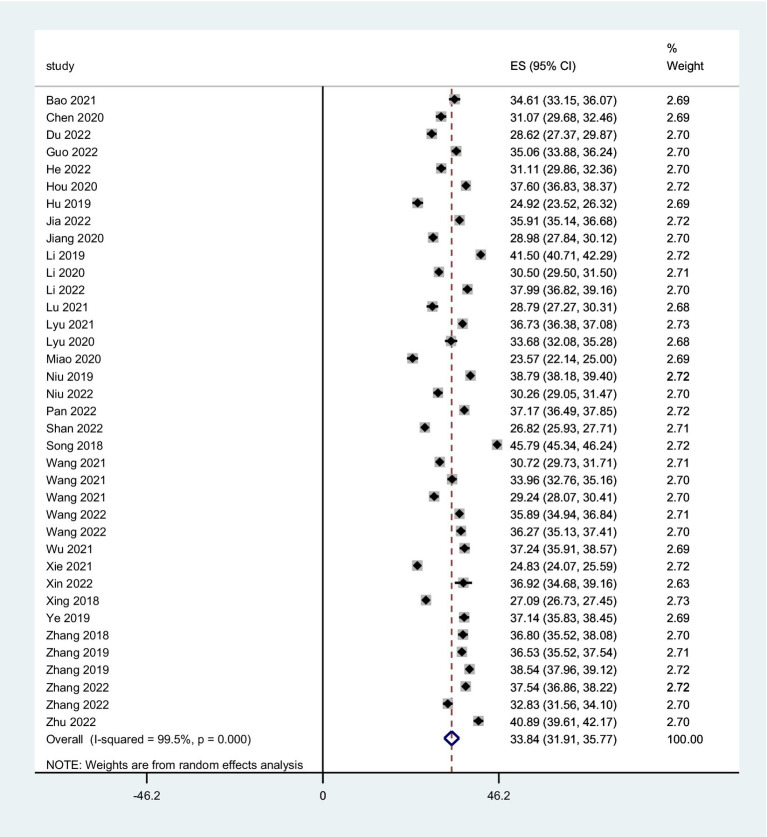
Forest plot of pooled mean scores for FoP.

**Figure 3:**
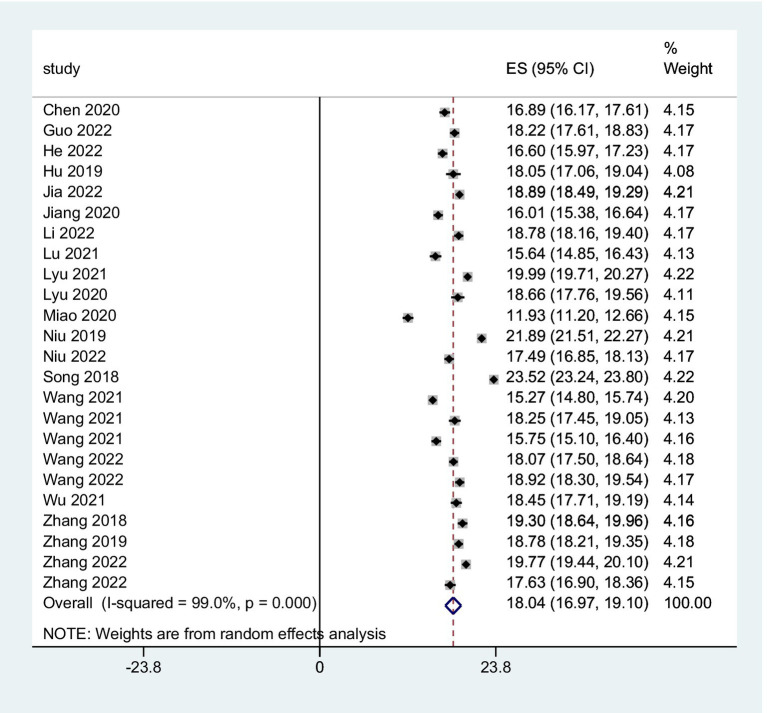
Forest plot of pooled mean scores for physiological health.

**Figure 4:**
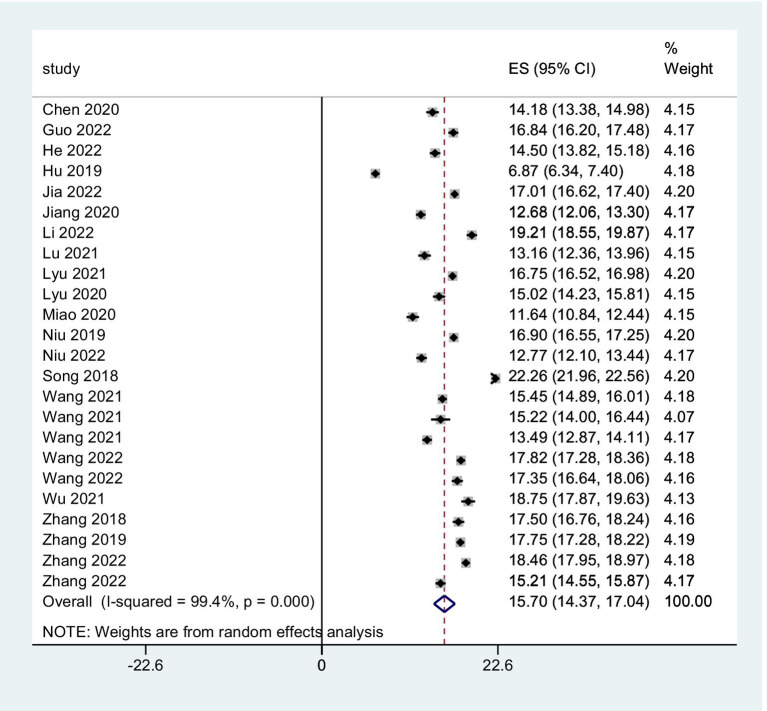
Forest plot of pooled mean scores for social/family health.

The combined FoP score of 37 studies resulted in 33.84 (95% *CI*: 31.91 ~ 35.77), with substantial heterogeneity (*Q* = 7099.06, *I*^2^ = 99.5%, *p* < 0.001) (Figure 2). The existence of substantial between-study variance is also reflected by the wide PI (21.57 ~ 46.11). Ten studies further analyzed two FoP dimensions: physiological health domain score was 18.04 (95% *CI*: 16.97 ~ 19.10) (Figure 3), with substantial heterogeneity (*Q* = 2304.27, *I*^2^ = 99.0%, *p* < 0.001), PI: 12.85 ~ 23.34; Social/family domain was 15.70 (95% *CI*: 14.37 ~ 17.04) (Figure 4), with substantial heterogeneity (*Q* = 3617.70, *I*^2^ = 99.4%, *p* < 0.001), PI: 9.30 ~ 22.19.

#### 3.4.2. Meta-regression analysis

Meta-regression uses regression equations to show how one or more variables affect the outcome variable. These variables may be study design, sample size, or case characteristics like patients’ mean age and height (Thompson and Sharp, 1999; Zhang et al., 2020). Based on prior studies (Kong et al., 2023; Nakamura et al., 2023), we assumed that heterogeneity could be caused by publication year, region, sample size, sample source, and literature quality. We performed meta-regression by the following covariates: publication year, region sample size (1–200, 200–400, more than 400), sample source (single-center, multicenter), and literature quality (medium quality, high quality). According to the findings, none of the covariates had a meaningful relationship with heterogeneity (*p* > 0.05), as shown in Table 2.

**Table 2:** A meta-regression analysis of included studies.

**Covariate**	***β***	***SE***	**95%*CI***	***P***
**Publication year**	−0.401	0.631	(−1.683, 0.880)	0.529
Region				
East China	−1.066	2.744	(−6.656, 4.524)	0.7
North China	−3.191	3.058	(−9.421, 3.038)	0.305
Central China	2.794	2.884	(−3.080, 8.667)	0.34
Northeast China	−3.967	5.460	(−15.089, 7.155)	0.473
Sample size				
1 ~ 200	−4.361	2.867	(−10.188, 1.466)	0.138
201 ~ 400	−1.829	2.744	(−7.404, 3.747)	0.510
**Sample source**	−0.439	2.17	(−4.845, 3.966)	0.841
**Literature quality**	0.328	1.916	(−3.562, 4.218)	0.865

#### 3.4.3. Subgroup analysis

We performed subgroup analysis by area, age, education level, marital status, place of residence, tumor stage, and recurrence and metastasis (see Table 3). According to the findings, the following patient subgroups had higher Fop scores: central China, under 45 years, junior high school or less, single, rural, stage IV, and with recurrence/metastasis. Each subgroup was subjected to the random-effects model because of the higher or moderate heterogeneity. Using sensitivity analysis, the origins of heterogeneity were further investigated. By excluding Zhu’s study (Zhu et al., 2022), heterogeneity in Central China decreased from 88.7 to 80.3%. The study’s participants were all post-radical mastectomy patients. The study subjects included in the study were all post-radical mastectomy patients. This may account for heterogeneity. After excluding Xie et al. (2021) from the study, the heterogeneity in the under 45 years group reduced from 94.8 to 85.9%. The study only included radiotherapy-treated breast cancer patients; therefore, heterogeneity may be related to treatment modality. After Shan et al. (2022) study was removed, the heterogeneity for this stage III subgroup dropped from 94.9 to 84.7%. Only first-episode breast cancer patients were included in the study. This may explain the heterogeneity. None of the remaining subgroup analyses used sensitivity analysis to discover the heterogeneity source. According to the literature, substantial heterogeneity may be associated with factors like the patient’s occupational status and disease duration.

**Table 3:** Subgroup analysis results of included studies.

Variable	Number of studies	Sample size	Overall effect	Effect size	Heterogeneity test
			Mean score(95%*CI*)	*Z*(*P*)	*I*^2^(*P*)
Region					
Eastern China	15	3,763	33.12 (29.33, 36.92)	17.11(<0.001)	99.6%(<0.001)
North China	8	1709	30.12 (26.92, 33.32)	18.47(<0.001)	98.6%(<0.001)
Central China	10	2,693	37.00 (36.22, 37.79)	92.71(<0.001)	88.7%(<0.001)
South China	5	1,267	32.59 (27.79, 37.38)	13.32(<0.001)	99.2%(<0.001)
Northeast China	1	257	30.26 (29.05, 31.47)	-	-
Age					
<45	16	1,193	35.68 (33.85, 37.50)	38.31(<0.001)	94.8%(<0.001)
≥45	14	1744	32.14 (30.06, 34.21)	30.38(<0.001)	97.6%(<0.001)
Education level					
Junior high school and below	17	2,109	35.21 (33.25, 37.17)	35.26(<0.001)	98.2%(<0.001)
High School	16	1,212	33.57 (31.78, 35.36)	36.82(<0.001)	97.1%(<0.001)
College and above	19	1,238	32.09 (30.26, 33.92)	34.42(<0.001)	96.4%(<0.001)
Marital status					
With spouse	19	3,998	34.06 (32.26, 35.87)	37.01(<0.001)	98.9%(<0.001)
No spouse	19	999	34.71 (32.92, 36.50)	37.95(<0.001)	95.2%(<0.001)
Place of residence					
Cities and towns	8	1,308	33.56 (31.43, 35.68)	30.96(<0.001)	97.6%(<0.001)
Rural	8	714	36.10 (34.32, 37.89)	39.7(<0.001)	93.2%(<0.001)
Disease stage					
I	10	419	34.49 (32.36, 36.62)	31.73(<0.001)	92.3%(<0.001)
II	10	850	35.81 (34.60, 37.02)	57.90(<0.001)	89.5%(<0.001)
III	14	1,126	37.98 (36.26, 39.71)	43.12(<0.001)	94.9%(<0.001)
IV	9	576	40.45 (36.72, 44.19)	21.25(<0.001)	97.9%(<0.001)
Recurrence/metastasis					
Yes	6	637,985	39.42 (36.62, 42.22)	27.59(<0.001)	96.2%(<0.001)
No	6		35.57 (33.05, 38.09)	27.64(<0.001)	97.5%(<0.001)

“-” means no such content.

### 3.5. Sensitivity and publication bias analysis

**Figure 5:**
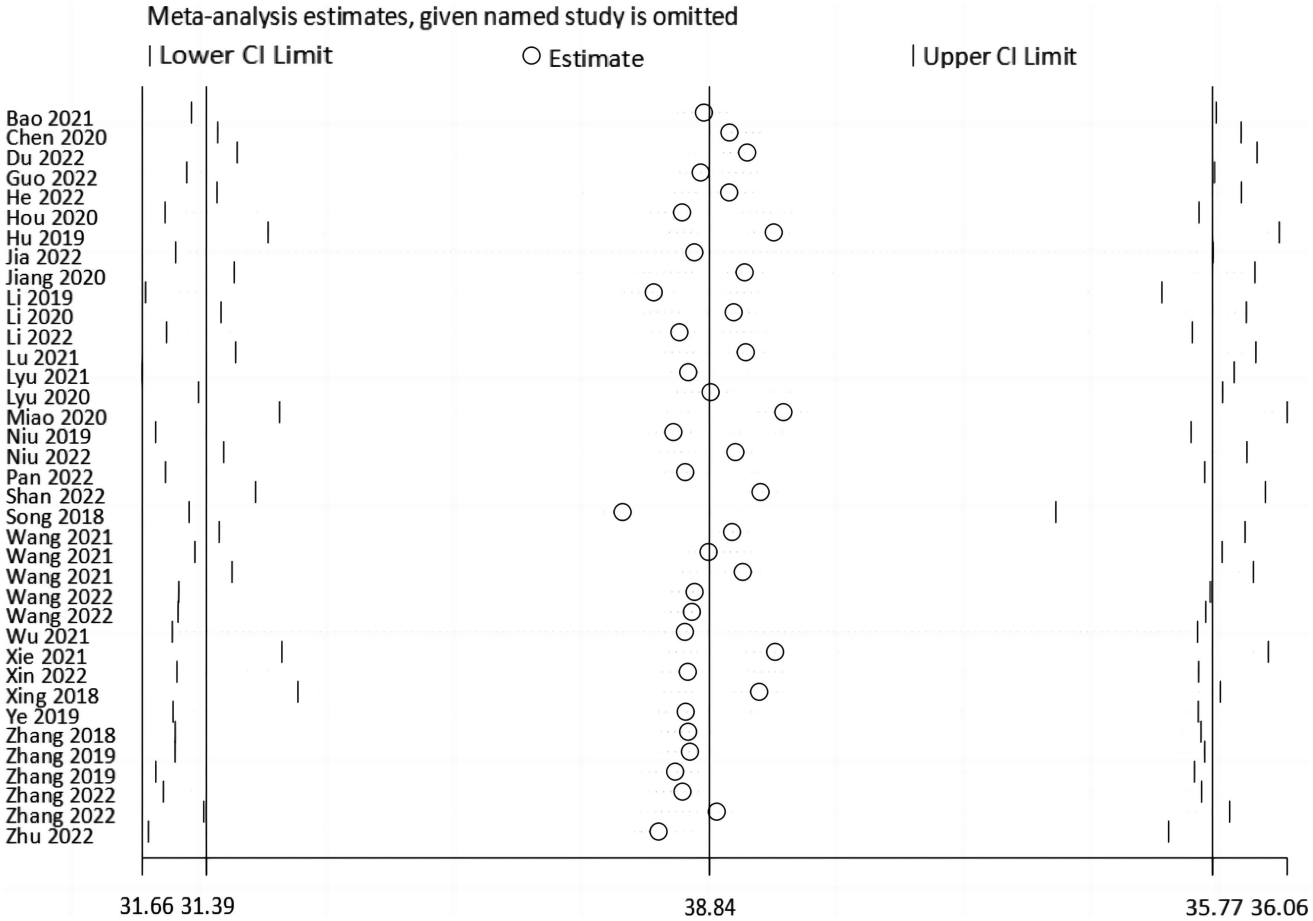
Sensitivity Analysis.

**Figure 6:**
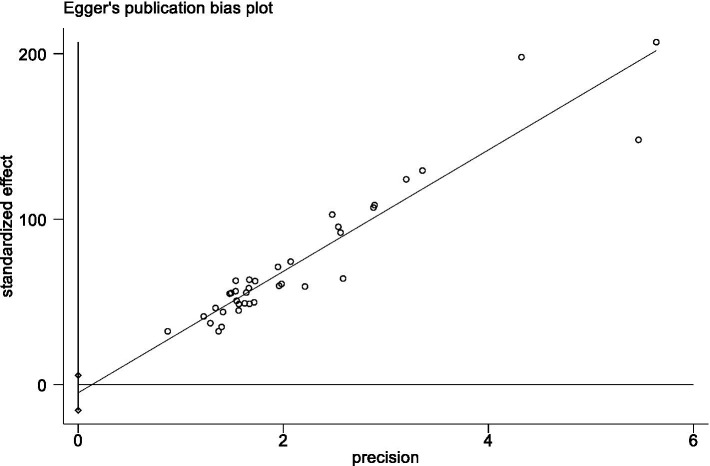
Publication bias analysis.

Sensitivity analyses were done by deleting specific studies one at a time. The sensitivity analysis revealed that the analysis’s results remained stable after excluding any single piece of literature, with the pooled mean of all the outcomes ranging from 33.51 (95% *CI*: 31.84 ~ 35.17) to 34.12 (95% *CI*: 32.19 ~ 36.06) (Figure 5). The Egger’s test result for the Fop score in breast cancer patients was −0.95 (*p* = 0.347), indicating that no publication bias existed (Figure 6).

## 4. Discussion

For future healthcare policy and FoP research, breast cancer patients’ FoP prevalence must be assessed. Our research will help estimate FoP’s burden and determine appropriate remedies for those in need. To our knowledge, this is the first large-scale study on the prevalence of Fop in Chinese breast cancer patients, and it will help advance our theoretical understanding of FoP in this population. This research examined 10 national and international databases and included 37 cross-sectional studies that met inclusion and exclusion criteria, a two-person screening, and a quality rating. Regarding research quality, 10 of this literature had an AHRQ score of 8, while the remaining 10 received a score of 5 to 7. Then, we conducted meta-regression and subgroup analyses to explore the source of heterogeneity. Additionally, we used sensitivity analysis to investigate the stability of study results and Egger’s test to evaluate publication bias in the included papers. This study also advises healthcare providers on reducing FoP in breast cancer patients quickly and effectively.

### 4.1. Overall levels of FoP in breast cancer patients

FoP is a common concern for cancer patients, and it has received more attention from psychology researchers. Several studies have shown that FoP can damage cancer survivors’ physiological, psychological, and social rehabilitative functions, affecting their prognosis and quality of life (Mehnert et al., 2009; Lane et al., 2019; Cui et al., 2022). This study’s meta-analysis revealed that Chinese breast cancer patients had a high FoP score of 33.84 (95% *CI*: 31.91 ~ 35.77), higher than developed countries like Germany (Mehnert et al., 2009; Koch-Gallenkamp et al., 2016). This may be due to the earlier focus on FoP in cancer patients in developed countries and the relatively sophisticated interventions they have in place to reduce the level of fear in patients. Furthermore, the findings of this study revealed that Chinese breast cancer patients had higher FoP levels than patients with other Chinese types of malignancies (Qiu and Yang, 2020; Hu et al., 2022). This may be because this breast cancer study exclusively included women. Psychological disorders like anxiety are more common in women than men (Hinz et al., 2010). This is also supported by previous studies for FoP (Mehnert et al., 2013). Additionally, this might be connected to the unique characteristics of the breast itself. The breast is a significant secondary female sex feature. After a breast cancer diagnosis, the patient’s body image and sense of self-society are easily impacted, making it harder for the patient to adequately mobilize available resources to cope with unfavorable occurrences and more prone to poor psychological adjustment (Quintard et al., 2014).

According to the findings of this study, the physiological health domain scored higher (18.04) than the social/family domain (15.70) on the two dimensions of the Fop scale. Patients’ fear of physiological health was greater than their fear of social/family domains, indicating that the symptoms of breast cancer disease and treatment are more intense, causing physical discomfort and significant harm to patients’ psychological health, resulting in fear and psychological burden. Therefore, healthcare professionals should focus on FoP levels in breast cancer patients and adjust their interventions accordingly. More intervention strategies for FoP in breast cancer patients are available, including mindfulness-based cognitive therapy (MBCT) (Hall et al., 2018), cognitively based compassion training (CBCT) (Gonzalez-Hernandez et al., 2018), and gratitude intervention (Otto et al., 2016). However, Chinese research on FoP in breast cancer survivors is primarily descriptive and lacks intervention studies. China can adapt foreign interventions to suit Chinese patients.

### 4.2. Regions

The subgroup analysis revealed that the FoP levels of breast cancer patients varied by region. The FoP levels of breast cancer patients in central China (37.00) were significantly higher than in other regions, which matched the findings of Li et al. (2023)study. According to certain research, the crude incidence rate of breast cancer in central China is the highest in the country (Shi et al., 2014), which may influence the FoP levels of patients in this region. Furthermore, the FoP levels of breast cancer patients may also be affected by regional differences in social customs, economic development, and health information (Herschbach et al., 2004; Kuang et al., 2022).

### 4.3. Ages

The findings of this study indicate a significant age-related difference in FoP levels among breast cancer patients. Like Mehnert et al. (2009) study findings, young and middle-aged breast cancer patients aged 45 years had significantly higher FoP levels (35.68) than those older than 45 years (32.14). Young and middle-aged patients are often healthy, making accepting a tumor’s sudden presence harder (Starreveld et al., 2018). Additionally, patients who are young or middle-aged have more obligations and duties in their personal and family lives, and they tend to avoid thinking about or talking about illness and death (Dong et al., 2021). Moreover, social experiences give people a lower psychological barrier to malignant tumors and experience more terror (Lim and Humphris, 2020). Healthcare personnel should pay attention to the psychological reconstruction of patients in this age group during treatment and nursing care, provide greater emotional and psychological support, and lessen their negative emotions and psychological burdens. Meanwhile, future research might examine the family and social factors that affect FoP in young breast cancer patients, perform a large-sample multicenter study, and apply applicable interventions to reduce FoP, enhancing patients’ prognosis and quality of life.

### 4.4. Education levels

Patients with various levels of education displayed several different levels of fear. According to the study’s findings, breast cancer patients with greater levels of education also had lower FoP levels, which is consistent with (Su et al. (2023) findings. Higher-educated patients can better comprehend and assimilate the information they are given and better comprehend the disease. They can accurately perceive the numerous side effects that may arise during therapy. They are more likely to use their psychological adjustment to sensibly and confidently relieve themselves. However, patients with lesser literacy levels do not comprehend the disease’s onset, progression, and prognosis and therefore have lower acceptance and increased psychological pressure (Jing and Zhang, 2020). Thus, healthcare providers should educate low-literate individuals about health and disease. The approach should differ from person to person, and the manner of education should be flexible. For instance, the Teach-back method (Ahmadidarrehsima et al., 2020) or animated educational movies can be used to assist patients in better comprehending and learning information about their sickness. This will lessen their sense of ambiguity about the condition and ultimately lower their FoP.

### 4.5. Marital statuses

This study validated (Kim and Kim (2022) findings that spouseless breast cancer patients had higher FoP levels. Single women are more likely to be concerned about how cancer would affect their fertility, marriage, and secondary sexual traits (Shrout et al., 2021). Additionally, unmarried female patients were more likely to experience anxieties because they lacked support from their partners, childcare, and emotional support. Su et al. (2023) research, however, produced different results. This might be because all the study participants in this investigation were female (Mehnert et al., 2013). Effective communication between breast cancer patients’ spouses can encourage and support them, helping them cope with the disease’s side effects (Moran et al., 2017). Therefore, patients’ spouses can be included in implementing nursing measures, and couple-centered psychological therapies can be adopted to boost patients’ psychological health. Medical staff should also understand the psychological concerns and requirements of unmarried young female patients and develop and implement specialized nursing interventions, like reproductive function protection. Simultaneously, medical personnel should encourage patients to speak with family members or friends to receive encouragement and support and to boost their confidence in surviving sickness.

### 4.6. Place of residence

Our findings demonstrate that FoP levels are higher in rural breast cancer patients. Location is another factor that influences the levels of FoP in breast cancer patients. Rural patients may have less access to health information than urban patients, and their anxieties and misconceptions about cancer may contribute to this (Ruan et al., 2020). Additionally, most patients in rural locations have very low-income levels, and the high costs of medical care could severely strain these families’ finances. This suggests that primary care clinicians spread breast cancer awareness, monitor FoP levels in female breast cancer patients, and implement the necessary interventions quickly.

### 4.7. Disease statuses

Like Dong et al. (2021) study, the current investigation discovered variations in FoP among female breast cancer patients with various cancer stages. Patients with stage IV breast cancer had significantly greater FoP levels than those with stages I, II, and III. A higher tumor stage may indicate greater malignancy, a worse chance of survival, a heavier burden of somatic symptoms, and a greater propensity for metastasis and recurrence (Hall et al., 2017). Therefore, patients are more prone to experience fear since they are under more psychological stress. Moreover, recurrent/metastatic breast cancer patients have higher FoP scores due to breast cancer’s metastasis and dissemination to nearby tissues or distant organ systems. Recurrent disease is the main reason breast cancer fails to improve long-term survival significantly (Soni et al., 2015), and when patients are aware of recurrence or metastasis, they can show signs of concern. This could impact patient adherence and cause some patients to lose faith in their treatment’s efficacy and stop receiving it. To reduce FoP, it is essential to enhance breast cancer treatment and control, shorten the disease’s progression, and adopt individualized care measures for advanced patients.

## 5. Limitations

There were several limitations in this study. First, our study only included Chinese breast cancer survivors; therefore, the findings may not apply to other nations or locations. Second, our meta-analysis was highly heterogenous and failed to identify its causes using meta-regression. Surgical procedures, radiation regimens, and disease stages may cause variations. We could not undertake additional subgroup analysis because none of the included studies provided detailed patient information. In future investigations, more comprehensive baseline data must be collected. Finally, the cross-sectional studies in this review did not capture the dynamic changes in FoP levels at different illness stages to provide a basis for intervention study cut-off times. In the future, our team will try to perform novel research. Nonetheless, we did our best to incorporate all relevant studies and hope our findings will be valuable for future clinical decision-making and research in breast cancer patients.

## 6. Conclusion

In conclusion, this study found higher FoP scores among Chinese breast cancer patients. Thus, healthcare professionals should monitor breast cancer patients’ FoP levels. According to our findings, FoP levels varied among breast cancer patients with diverse regions, ages, educational levels, marital statuses, places of residence, disease stages, and disease statuses. Thus, to reduce patients’ FoP levels and improve their quality of life, healthcare practitioners should design timely and effective intervention techniques based on patient-specific situations. These findings imply that more studies should be conducted to discover which sociodemographic and disease-related variables most strongly affect FoP onset. This may offer the theoretical basis for FoP management and treatment.

## Data availability statement

The original contributions presented in the study are included in the article/Supplementary material, further inquiries can be directed to the corresponding authors.

## Author contributions

J-LH, JG, and Y-FY proposed the concept and supervised the work. JG and Y-FY designed the work and provided solutions for inconsistencies. J-LH, D-JH, and JY performed the literature search, study selection, and data extraction. H-QX, X-YG, and X-YL performed the data analysis and interpretation. J-LH, H-QX, and JY wrote the manuscript, and contributed to the article in the same way. Also, WW and M-JC assisted in the review and checking of the manuscript. All authors contributed to the article and approved the submitted version.

## Conflict of interest

The authors declare that the research was conducted in the absence of any commercial or financial relationships that could be construed as a potential conflict of interest.

## Publisher’s note

All claims expressed in this article are solely those of the authors and do not necessarily represent those of their affiliated organizations, or those of the publisher, the editors and the reviewers. Any product that may be evaluated in this article, or claim that may be made by its manufacturer, is not guaranteed or endorsed by the publisher.
