# Supplementary material for: Fear of disease progression among breast cancer patients in China: a meta-analysis of studies using the fear of progression questionnaire short form
Source: Front Psychol. 2023 Aug 23;14:1222798. doi: 10.3389/fpsyg.2023.1222798 (PMC10482266; doi:10.3389/fpsyg.2023.1222798)
Supplement: Supplementary file 1 [file Table_1.DOCX]

**Supplementary Materials**

Appendix A: Search strategies of each database.

Appendix B: Methodological quality appraisal results based on the AHRQ tool for each study.

**Appendix A**

**1.PubMed**

| #1 | ("Breast Neoplasms"[Mesh]) OR ("Breast Carcinoma in Situ"[Mesh]) |
| --- | --- |
| #2 | (Breast Neoplasm*[Title/Abstract]) OR (Breast Tumor*[Title/Abstract]) OR (Breast Cancer[Title/Abstract]) OR (Mammary Cancer*[Title/Abstract]) OR (Malignant Neoplasm of Breast[Title/Abstract]) OR (Breast Malignant Neoplasm*[Title/Abstract]) OR (Malignant Tumor of Breast[Title/Abstract]) OR (Breast Malignant Tumor[Title/Abstract]) OR (Cancer of Breast[Title/Abstract]) OR (Human Mammary Carcinoma*[Title/Abstract]) OR (Human Mammary Neoplasm*[Title/Abstract]) OR (Breast Carcinoma*[Title/Abstract]) OR (Mammary Carcinoma*[Title/Abstract]) |
| #3 | #1 OR #2 |
| #4 | ("Fear"[Mesh]) |
| #5 | (worry[Title/Abstract]) OR (concern[Title/Abstract]) OR (uncertainty[Title/Abstract]) OR (anxiety[Title/Abstract]) |
| #6 | #4 OR #5 |
| #7 | ("recurrence"[Mesh]) OR ("Neoplasm Recurrence, Local"[Mesh]) OR ("Disease Progression"[Mesh]) |
| #8 | (Recurrenc*[Title/Abstract]) OR (Recrudescence[Title/Abstract]) OR (uncertainty[Title/Abstract]) OR (Progression, Disease[Title/Abstract]) OR (Clinical Course[Title/Abstract]) OR (Clinical Progression[Title/Abstract]) OR (Progression, Clinical[Title/Abstract]) OR (Disease Exacerbation[Title/Abstract]) OR (Exacerbation, Disease[Title/Abstract]) OR (Relapse[Title/Abstract]) |
| #9 | #7 OR #8 |
| #10 | (Epidemiology[MeSH]) OR (Incidence[MeSH]) OR (Prevalence[MeSH]) |
| #11 | (epidemi*[Title/Abstract]) OR (frequency[Title/Abstract]) OR (surveillance[Title/Abstract]) OR (occurrence[Title/Abstract]) OR (outbreaks[Title/Abstract]) OR (incidences[Title/Abstract]) OR (Risk Factor*[Title/Abstract]) OR (Related Factor*[Title/Abstract]) OR (Influencing Factor*[Title/Abstract]) OR (Affecting Factor*[Title/Abstract]) OR (Influe*[Title/Abstract]) OR (Population* at Risk[Title/Abstract]) |
| #12 | #10 OR #11 |
| #13 | ("China"[Mesh]) OR ("Taiwan"[Mesh]) |
| #14 | (China[Text Word]) OR (Chinese[Text Word]) OR (People's Republic of China[Text Word]) OR (Mainland China[Text Word]) OR (Manchuria[Text Word]) OR (Sinkiang[Text Word]) OR (Inner Mongolia[Text Word]) OR (Beijing[Text Word]) OR (Hong Kong[Text Word]) OR (Macau[Text Word]) OR (Tibet[Text Word]) OR (Taiwan[Text Word]) |
| #15 | #13 OR #14 |
| #10 | #3 AND #6 AND #9 AND #12 AND #15 |

**2.Web of Science**

| #1 | TOPIC: (Breast Neoplasm* OR Breast Neoplasms in Situ OR Breast Tumor* OR Breast Cancer OR Mammary Cancer* OR Malignant Neoplasm of Breast OR Breast Malignant Neoplasm* OR Malignant Tumor of Breast OR Breast Malignant Tumor OR Cancer of Breast OR Human Mammary Carcinoma* OR Human Mammary Neoplasm* OR Breast Carcinoma* OR Mammary Carcinoma*) |
| --- | --- |
| #2 | TOPIC:( Fear OR worry OR concern OR uncertainty OR anxiety) |
| #3 | TOPIC:(Recurrence OR Neoplasm Recurrence, Local OR Disease Progression Recurrenc* OR Recrudescence OR Progression, Disease OR Clinical Course OR Clinical Progression OR Progression, Clinical OR Disease Exacerbation OR Exacerbation, Disease OR Relapse) |
| #4 | TOPIC:(Epidemiology OR Incidence OR Prevalence OR Risk Factors OR frequency OR surveillance OR occurrence OR outbreaks OR incidences OR Risk Factor OR Related Factor OR Influencing Factor OR Affecting Factor OR Population at Risk OR Influe*) |
| #5 | CU：(China OR Taiwan OR Taiwanese OR Chinese OR Mainland China OR  Manchuria OR Sinkiang OR Inner Mongolia OR Macau OR Tibet) |
| #6 | #1 AND #2AND #3 AND #4 AND #5 |

**3.EMBASE**

| #1 | 'breast cancer'/exp OR 'benign breast tumor'/exp |
| --- | --- |
| #2 | 'breast cancer':ab,ti OR 'breast gland tumor':ab,ti OR 'breast gland tumour':ab,ti OR 'breast mass':ab,ti OR 'breast neoplasms':ab,ti OR 'breast tumour':ab,ti OR 'breast neoplasm':ab,ti OR 'mamma tumor':ab,ti OR 'mamma tumour':ab,ti OR 'mammary gland tumour':ab,ti OR 'mammary neoplasms':ab,ti OR 'mammary tumor':ab,ti OR 'mammary tumor cell':ab,ti OR 'mammary tumour':ab,ti OR 'mammary tumour cell':ab,ti OR 'unilateral breast neoplasms':ab,ti |
| #3 | #1 OR #2 |
| #4 | 'Fear'/exp OR 'Anxiety'/exp |
| #5 | 'worry':ab,ti OR 'concern':ab,ti OR 'uncertainty':ab,ti |
| #6 | #4 OR #5 |
| #7 | 'cancer recurrence'/exp OR 'recurrent disease'/exp |
| #8 | 'Neoplasm Recurrence, Local':ab,ti OR 'Disease Progression Recurrenc*':ab,ti OR 'Recrudescence':ab,ti OR 'Progression, Disease':ab,ti OR 'Clinical Progression':ab,ti OR 'Relapse':ab,ti |
| #9 | #7 OR #8 |
| #10 | 'Epidemiology'/exp OR 'Incidence'/exp OR 'Prevalence'/exp OR 'Risk Factors'/exp |
| #11 | 'frequency':ab,ti OR 'surveillance':ab,ti OR 'occurrence':ab,ti OR 'outbreaks':ab,ti OR 'incidences':ab,ti OR 'Risk Factor':ab,ti OR 'Related Factor':ab,ti OR 'Influencing Factor':ab,ti OR 'Affecting Factor':ab,ti OR 'Population at Risk':ab,ti OR 'Influe*':ab,ti |
| #12 | #10 OR #11 |
| #13 | 'China'/exp OR 'Taiwan'/exp |
| #14 | Taiwan or Taiwanese or China or Chinese or 'Mainland China' or Manchuria or Sinkiang or 'Inner Mongolia' or 'Hong Kong' or Macau or Tibet):ab,ti |
| #15 | #13 OR #14 |
| #16 | #3 AND #6 AND #9 AND #12 AND #15 |

**4.Cochrane Library Databases**

| #1 | Breast Neoplasm* OR Breast Neoplasms in Situ OR Breast Tumor* OR Breast Cancer OR Mammary Cancer* OR Malignant Neoplasm of Breast OR Breast Malignant Neoplasm* OR Malignant Tumor of Breast OR Breast Malignant Tumor OR Cancer of Breast OR Human Mammary Carcinoma* OR Human Mammary Neoplasm* OR Breast Carcinoma* OR Mammary Carcinoma*:ti,ab,kw |
| --- | --- |
| #2 | Fear OR worry OR concern OR uncertainty OR anxiety:ti,ab,kw |
|  | Recurrence OR Neoplasm Recurrence, Local OR Disease Progression Recurrenc* OR Recrudescence OR Progression, Disease OR Clinical Course OR Clinical Progression OR Progression, Clinical OR Disease Exacerbation OR Exacerbation, Disease OR Relapse:ti,ab,kw |
| #3 | Epidemiology OR Incidence OR Prevalence OR Risk Factors OR frequency OR surveillance OR occurrence OR outbreaks OR incidences OR Risk Factor OR Related Factor OR Influencing Factor OR Affecting Factor OR Population at Risk OR Influe*:ti,ab,kw |
| #4 | China OR Taiwan OR Taiwanese OR Chinese OR "Mainland China" OR  Manchuria OR Sinkiang OR "Inner Mongolia" OR Macau OR Tibet:tx |
| #5 | #1 AND #2AND #3AND #4 |

**5.CINAHL**

| #1 | TI Breast Neoplasm* OR AB Breast Neoplasms in Situ OR AB Breast Tumor* OR AB Breast Cancer OR AB Mammary Cancer* OR AB Malignant Neoplasm of Breast OR AB Breast Malignant Neoplasm* OR AB Malignant Tumor of Breast OR AB Breast Malignant Tumor OR AB Cancer of Breast OR AB Human Mammary Carcinoma* OR AB Human Mammary Neoplasm* OR AB Breast Carcinoma* OR AB Mammary Carcinoma* |
| --- | --- |
| #2 | AB Fear OR AB worry OR AB concern OR AB uncertainty OR AB anxiety |
| #3 | AB Recurrence OR AB Neoplasm Recurrence, Local OR AB Disease Progression Recurrenc* OR AB Recrudescence OR AB Progression, Disease OR AB Clinical Course OR AB Clinical Progression OR AB Progression, Clinical OR AB Disease Exacerbation OR AB Exacerbation, Disease OR AB Relapse |
| #4 | AB Epidemiology OR AB Incidence OR AB Prevalence OR AB Risk Factors OR AB Frequency OR AB Surveillance OR AB Occurrence OR AB Outbreaks OR AB Incidences OR AB Risk Factor OR AB Related Factor OR AB Influencing Factor OR AB AffecABng Factor OR AB Population at Risk OR AB Influe* |
| #5 | TX China OR Taiwan OR TX Taiwanese OR TX Chinese OR TX Mainland China OR TX Manchuria OR TX Sinkiang OR TX Inner Mongolia OR TX Macau OR TX Tibet |
| #6 | #1 AND #2AND #3 AND #4AND #5 |

**6.PsycINFO**

| #1 | (TI Breast Neoplasm* OR AB Breast Neoplasms in Situ OR AB Breast Tumor* OR AB Breast Cancer OR AB Mammary Cancer* OR AB Malignant Neoplasm of Breast OR AB Breast Malignant Neoplasm* OR AB Malignant Tumor of Breast OR AB Breast Malignant Tumor OR AB Cancer of Breast OR AB Human Mammary Carcinoma* OR AB Human Mammary Neoplasm* OR AB Breast Carcinoma* OR AB Mammary Carcinoma*) |
| --- | --- |
| #2 | AB Fear OR AB worry OR AB concern OR AB uncertainty OR AB anxiety |
| #3 | AB Recurrence OR AB Neoplasm Recurrence, Local OR AB Disease Progression Recurrenc* OR AB Recrudescence OR AB Progression, Disease OR AB Clinical Course OR AB Clinical Progression OR AB Progression, Clinical OR AB Disease Exacerbation OR AB Exacerbation, Disease OR AB Relapse |
| #4 | AB Epidemiology OR AB Incidence OR AB Prevalence OR AB Risk Factors OR AB Frequency OR AB Surveillance OR AB Occurrence OR AB Outbreaks OR AB Incidences OR AB Risk Factor OR AB Related Factor OR AB Influencing Factor OR AB AffecABng Factor OR AB Population at Risk OR AB Influe* |
| #5 | TX China OR Taiwan OR TX Taiwanese OR TX Chinese OR TX Mainland China OR TX Manchuria OR TX Sinkiang OR TX Inner Mongolia OR TX Macau OR TX Tibet |
| #6 | #1 AND #2AND #3 AND #4AND #5 |

**7.WanFang Database**

| #1 | 主题:("乳腺癌" or "乳腺肿瘤" or "乳腺癌症" or "乳癌" or "乳腺瘤") |
| --- | --- |
| #2 | 主题:("恐惧" or "担忧" or "不确定感") |
| #3 | 主题:("复发" or "疾病进展" ) |
| #4 | 主题:("患病率"or"发病率"or"流行病学"or"流行病学研究"or"现患调查"or"发病率研究"or"患病率"or"危险因素"or"影响因素"or"相关因素"or"相关性研究"or"相关因素分析"or"影响") |
| #5 | #1 AND #2 AND #3 AND #4 |

**8.China Knowledge Resource Integrated Database(CNKI)**

| #1 | SU=('乳腺癌'+'乳腺肿瘤'+'乳腺癌症'+'乳癌'+'乳腺瘤') |
| --- | --- |
| #2 | SU= ('恐惧'+ '担忧'+'不确定感') |
| #3 | SU= ('复发'+ '疾病进展') |
| #4 | SU=('患病率'+'发病率'+'流行病学'+'流行病学研究'+'现患调查'+'发病率研究'+'发生率'+'危险因素'+'影响因素'+'相关因素'+'相关性研究'+'相关因素分析'+'影响') |
| #5 | #1 AND #2 AND #3 AND #4 |

**9.Chinese Biomedical Database(CBM)**

| #1 | "乳腺癌"[常用字段:智能] OR "乳腺肿瘤"[常用字段:智能] OR "乳癌"[常用字段:智能] OR "乳腺瘤"[常用字段:智能] OR "乳腺癌症"[常用字段:智能] |
| --- | --- |
| #2 | "恐惧"[常用字段:智能] OR "担忧"[常用字段:智能] OR "不确定感"[常用字段:智能] |
| #3 | "复发"[常用字段:智能] OR "疾病进展"[常用字段:智能] |
| #4 | "患病率"[常用字段:智能] OR "发病率"[常用字段:智能] OR "流行病学"[常用字段:智能] OR "流行病学研究"[常用字段:智能] OR "现患调查"[常用字段:智能] OR "发病率研究"[常用字段:智能] OR "发生率"[常用字段:智能] OR "危险因素"[常用字段:智能] OR "影响因素"[常用字段:智能] OR "相关因素"[常用字段:智能] OR "相关性研究"[常用字段:智能] OR "相关因素分析"[常用字段:智能] OR "影响"[常用字段:智能] |
| #5 | #1 AND #2 AND #3 AND #4 |

**10.Chinese Science and Technology Periodicals(VIP)**

| #1 | 题名和关键词:乳腺癌 or 乳腺肿瘤 or 乳腺癌症 or 乳癌 or 乳腺瘤 |
| --- | --- |
| #2 | 题名和关键词:恐惧 or 担忧 or 不确定感 |
| #3 | 题名和关键词:复发 or 疾病进展 |
| #4 | 摘要:患病率 or 发病率 or 流行病学 or 流行病学研究 or 现患调查 or 发病率研究 or患病率 or 危险因素 or 影响因素 or 相关因素 or 相关性研究 or 相关因素分析 or 影响 |
| #5 | #1 AND #2 AND #3 AND #4 |

**Appendix B**

| Table 1Methodological quality appraisal results based on the AHRQ tool for each study. | | | | | | | | | | | | | |
| --- | --- | --- | --- | --- | --- | --- | --- | --- | --- | --- | --- | --- | --- |
| Stady | Item 1 | Item 2 | Item 3 | Item 4 | Item 5 | Item 6 | Item 7 | Item 8 | Item 9 | Item 10 | Item 11 | Total score | Quality |
| Bao 2021 | Y | Y | Y | Y | Y | N | N | N | N | Y | U | 6 | M |
| Chen 2020 | Y | Y | Y | Y | Y | N | N | Y | N | Y | U | 7 | M |
| Du 2022 | Y | Y | Y | Y | Y | N | Y | Y | N | Y | U | 8 | H |
| Guo 2022 | Y | Y | Y | Y | Y | Y | Y | N | N | Y | U | 8 | H |
| He 2022 | Y | Y | Y | Y | Y | N | N | N | N | Y | U | 6 | M |
| Hou 2020 | Y | N | Y | Y | Y | N | N | Y | N | N | U | 5 | M |
| Hu 2019 | Y | Y | Y | Y | Y | N | N | Y | N | Y | U | 7 | M |
| Jia 2022 | Y | Y | Y | Y | Y | N | N | Y | N | Y | U | 7 | M |
| Jiang 2020 | Y | Y | Y | Y | Y | N | N | N | N | Y | U | 6 | M |
| Li 2019 | Y | Y | Y | Y | Y | N | N | Y | N | Y | U | 7 | M |
| Li 2021 | Y | Y | Y | Y | Y | N | N | N | N | Y | U | 6 | M |
| Li 2022 | Y | Y | Y | Y | Y | Y | N | Y | N | Y | Y | 8 | H |
| Lu 2021 | Y | Y | Y | Y | Y | Y | N | Y | N | Y | U | 8 | H |
| Lyu 2021 | Y | Y | Y | Y | Y | N | N | Y | N | N | U | 6 | M |
| Lyu 2020 | Y | Y | Y | Y | Y | N | Y | Y | N | Y | N | 8 | H |
| Miao 2020 | Y | Y | Y | Y | Y | N | N | N | N | Y | U | 6 | M |
| Niu 2019 | Y | Y | Y | Y | Y | N | Y | Y | N | Y | U | 8 | H |
| Niu 2022 | Y | Y | Y | Y | Y | Y | N | Y | N | Y | U | 8 | H |
| Pan 2022 | Y | Y | Y | Y | Y | N | N | Y | N | Y | U | 7 | M |
| Shan 2022 | Y | Y | Y | Y | Y | N | N | Y | N | Y | U | 7 | M |
| Song 2018 | Y | Y | Y | Y | Y | N | N | Y | N | Y | U | 7 | M |
| Wang 2021 | Y | Y | Y | Y | Y | N | N | Y | N | Y | U | 6 | M |
| Wang 2021 | Y | Y | Y | Y | Y | N | N | N | N | Y | U | 6 | M |
| Wang 2021 | Y | Y | Y | Y | Y | Y | N | Y | N | Y | U | 8 | H |
| Wang 2022 | Y | Y | Y | Y | Y | N | N | N | N | Y | U | 6 | M |
| Wang 2022 | Y | Y | Y | Y | Y | N | N | Y | N | Y | U | 7 | M |
| Wu 2021 | Y | Y | Y | Y | Y | N | N | Y | N | Y | U | 7 | M |
| Xie 2021 | Y | Y | Y | Y | Y | N | N | Y | N | Y | U | 7 | M |
| Xin 2022 | Y | Y | Y | Y | Y | N | N | Y | N | Y | U | 7 | M |
| Xing 2018 | Y | Y | Y | Y | Y | N | N | Y | N | N | U | 6 | M |
| Ye 2019 | Y | Y | Y | Y | Y | N | N | Y | N | Y | U | 7 | M |
| Zhang 2018 | Y | Y | Y | Y | Y | Y | N | Y | N | N | U | 7 | M |
| Zhang 2019 | Y | Y | Y | Y | Y | N | N | Y | N | Y | U | 7 | M |
| Zhang 2019 | Y | Y | Y | Y | Y | N | N | Y | N | Y | U | 7 | M |
| Zhang 2022 | Y | Y | Y | Y | Y | Y | N | Y | N | Y | U | 8 | H |
| Zhang 2022 | Y | Y | Y | Y | Y | Y | N | Y | N | Y | U | 8 | H |
| Zhu 2022 | Y | Y | Y | Y | Y | N | N | Y | N | Y | U | 7 | M |
| Y: yes; N: no; U: unclear; H: high quality; M: medium quality.  Item 1: Define the source of information (survey, record review).  Item 2: List inclusion and exclusion criteria for exposed and unexposed subjects (cases and controls) or refer to previous publications.  Item 3: Indicate time period used for identifying patients.  Item 4: Indicate whether or not subjects were consecutive if not population-based.  Item 5: Indicate if evaluators of subjective components of study were masked to other aspects of the status of the participants.  Item 6: Describe any assessments undertaken for quality assurance purposes (e.g., test/retest of primary outcome measurements).  Item 7: Explain any patient exclusions from analysis.  Item 8: Describe how confounding was assessed and/or controlled.  Item 9: If applicable, explain how missing data were handled in the analysis.  Item 10: Summarize patient response rates and completeness of data collection.  Item 11: Clarify what follow-up, if any, was expected and the percentage of patients for which incomplete data or follow-up was obtained. | | | | | | | | | | | | | |
